# Did a Nocebo Effect Contribute to the Rise in Special Education Enrollment Following the Flint, Michigan Water Crisis?

**DOI:** 10.32872/cpe.9577

**Published:** 2023-03-31

**Authors:** Siddhartha Roy, Keith J. Petrie, Greg Gamble, Marc A. Edwards

**Affiliations:** 1 Department of Civil and Environmental Engineering, Virginia Tech, Blacksburg, VA, USA; 2 UNC Water Institute, Gillings School of Global Public Health, University of North Carolina, Chapel Hill, NC, USA; 3 Department of Psychological Medicine, University of Auckland, Auckland, New Zealand; 4 Department of Medicine, University of Auckland, Auckland, New Zealand; Philipps-University of Marburg, Marburg, Germany

**Keywords:** blood lead, lead exposure, Flint Water Crisis, nocebo effect, special education

## Abstract

**Background:**

Exposure to waterborne lead during the Flint Water Crisis during April 2014-October 2015 is believed to have caused increased special education enrollment in Flint children.

**Method:**

This retrospective population-based cohort study utilized de-identified data for children under six years of age who had their blood lead tested during 2011 to 2019, and special education outcomes data for children enrolled in public schools for corresponding academic years (2011-12 to 2019-20) in Flint, Detroit (control city) and the State of Michigan. Trends in the following crisis-related covariates were also evaluated: waterborne contaminants, poverty, nutrition, city governance, school district policies, negative community expectations, media coverage and social media interactions.

**Results:**

Between 2011 and 2019, including the 2014-15 crisis period, the incidence of elevated blood lead in Flint children (≥ 5µg/dL) was always at least 47% lower than in the control city of Detroit (p < .0001) and was also never significantly higher than that for all children tested in Michigan (p = 0.33). Nonetheless, special education enrollment in Flint spiked relative to Detroit and Michigan (p < .0001). There is actually an inverse relationship between childhood blood lead and special education enrollment in Flint.

**Conclusion:**

This study failed to confirm any positive association between actual childhood blood lead levels and special education enrollment in Flint. Negative psychological effects associated with media predictions of brain damage could have created a self-fulfilling prophecy via a nocebo effect. The findings demonstrate a need for improved media coverage of complex events like the Flint Water Crisis.

## Highlights

- Waterborne lead exposure during the Flint crisis did not correlate with special education enrollment.
- Flint children were repeatedly labeled as lead poisoned and brain damaged in the aftermath.
- A nocebo effect could have contributed to negative educational outcomes in Flint.
- Erroneous, negative media labels can be internalized and lead to psychological harm in children.

In April 2014, the city of Flint, Michigan stopped purchasing treated Lake Huron water from Detroit and switched to corrosive Flint River water as a cost savings measure. The city also interrupted the addition of corrosion control chemicals to the treated water, which were required under federal regulations to reduce the leaching of the neurotoxin lead from lead pipes and home plumbing. This increased lead levels in tap water and children’s blood mainly in the months of June-August 2014 (Roy et al., 2019). In response to residents’ concerns, two of the authors assisted with sampling 269 Flint homes in 2015, proving the 90th percentile water lead level (27 μg/L) was almost twice the US Environmental Protection Agency (EPA) action level of 15 μg/L (Pieper et al., 2018). It was later revealed that the proportion of children < 6 years of age with elevated blood lead, i.e., ≥ 5 μg/dL US Centers for Disease Control and Prevention (CDC) reference level, increased following the water switch (Hanna-Attisha et al., 2016), primarily in June-August 2014 (Roy et al., 2019). Michigan officials later announced a Legionnaire’s Disease outbreak that killed at least 13 people (Rhoads et al., 2017). These events became known in the media as the Flint Water Crisis (FWC). After the water problems were exposed, Flint reconnected to Detroit water in October 2015, a federal emergency was declared in January 2016, and over US$1.2 billion in relief funds have been appropriated for residents including free bottled water (through April 2018), free lead faucet filters, health interventions, settlement money for lead-exposed children, special education services, and replacement of around 12,000 lead pipes to be completed in 2023 (Bosman, 2020; City of Flint, 2022; Roy & Edwards, 2019a; Roy & Edwards, 2020). Flint water has met all federal standards since late 2016 and many residents still consume only bottled water due to lost trust (Flint Cares, 2018; Fonger et al., 2019; Reuben et al., 2022; Roy, 2017; Roy & Edwards, 2019b; Sobeck et al., 2020).

Recent media reports (see Supplementary Materials [SM] Table S1) attribute increasing rates of special education enrollment and diagnoses of learning disabilities in Flint children to lead exposure and “lead poisoning” from the FWC (Alfonsi, 2020; Green, 2019) but none of these conclusions are based on peer reviewed data. Blood lead levels have been steadily dropping in the United States and in Flint for the past 50 years following the banning of lead from gasoline, paint and pipes (Dignam et al., 2019; Gómez et al., 2018). The peak childhood blood lead levels during the FWC (2014-15) were well below those recorded in Flint during 2011 (Gómez et al., 2018; Roy et al., 2019).

In this study, we investigate the hypothesis that increased negative educational outcomes were caused by lead exposure from the FWC as has been stated by the media and experts (ACLU, 2016; Alfonsi, 2020; Green, 2019; Redlener, 2018; Riley, 2018; Strauss, 2019). Trends in blood lead levels of Flint children were compared to the control city of Detroit, which has comparable socioeconomic and racial make-up (Table 1) and also used the same drinking water for over 50 years except for the 18 months of the FWC. We also compare Flint to state-wide trends from Michigan, and evaluate relevant extraneous factors that may have affected educational outcomes in Flint children.

**Table 1:** Key Demographic Factors of Comparison for Flint and Detroit (Control City)

Measure	Flint	Detroit (Control)
Water source during:		
1950s – Apr 2014	Lake Huron	Lake Huron
Apr 2014 – Oct 2015	Flint River	Lake Huron
Oct 2015 – present	Lake Huron	Lake Huron
Approximate count of lead service line connections (% of total water connections)	Pre-2016: 12,000 (40%) Current: <1,400 (4.7%)	80,000 (40%)
Drinking water source in public schools	Bottled water (Sep 2015-Feb 2022) Filtered water (Feb 2022-present)	Bottled water Aug 2018-Aug 2019) Filtered water (Aug 2019-present)
Net change in population (2011 to 2019), %* ^#^	–8.4% (105,391 to 96,559)	–8.6% (738,223 to 674,841)
Population < 5 years old (range during 2011-19), %*	7.5-8.3%	7.0-7.3%
Persons per household, 2014-18	2.36	2.55
Net change in unemployment rate (2011 to 2019), %^†^	–52.1% (19% to 9.1%)	–58.1% (20.5% to 8.6%)
Net change in median household income (2011 to 2019), %*	+8.3% ($26,621 to $28,834)	+10.9% ($27,862 to $30,894)
Health outcomes (range during 2011-2019), overall rank in Michigan	77-82 of 83 (Genesee Co.)	81-83 of 83 (Wayne Co.)
Percent below poverty level (range during 2011-19), %*	38.8-41.9%	35-40.9%
Worst American city to live in, rank (based on 2015 data)	#1	#3
% decline in total students attending public schools in 10 years (2009-10 till 2018-19)	43.1%	68.4%
% of total resident students attending charter schools, 2018-19 (national rank in charter school enrollment)	45.6% (#3)	37.9% (#2)

*Data from American Community Survey 5-Year Estimates Data Profiles via US Census (US Census Bureau, 2022).

^#^US Census language: Estimates are not comparable to other geographic levels of health estimates (due to methodology differences that may exist between different data sources).

^†^Data from Michigan Bureau of Labor Market (Michigan Department of Technology Management and Budget, 2022).

Other data references: City of Flint, 2022; David et al., 2017; Goetz, 2022; Mack, 2019; Sauter et al., 2017; University of Wisconsin Population Health Institute, 2022.

After demonstrating that covariates unlikely played a primary role (see Text S1, Supplementary Materials), we probe the possibility of a nocebo effect (Barsky et al., 2002; Petrie & Rief, 2019) or a self-fulfilling prophecy, associated with repeated predictions of brain damage to Flint children via the intense publicity associated with the FWC. Research has shown that parents’ and teachers’ negative expectations of children can have adverse effects on educational outcomes. Past studies also suggest that these effects are cumulative and have a greater impact on disadvantaged populations (Jussim et al., 2009; Madon et al., 1997; Madon et al., 2011; Rosenthal & Jacobson, 1968). To examine the interaction between media stories and community perceptions, we evaluated a) representative national and local media stories and associated social media interactions, b) public statements of government, medical and school leaders, and c) resident feedback in media’s news stories, highlighting the purported effects of lead and “lead poisoning” during the FWC period on children and their educational outcomes in Flint and the control city of Detroit.

## Materials and Method

### Elevated Blood Lead

Childhood blood lead testing is required under Medicaid, where all children receive a screening blood lead test at ages 1 and 2 years, and up to 5 years (Cantor et al., 2019; US Preventive Services Task Force et al., 2019), but not all children receive such tests in practice. The State of Michigan sampling methodology and reporting guidelines have not changed markedly since 1998 (Michigan Department of Health and Human Services, 2020). The percentage of children under six years of age with blood lead above the 2012-21 CDC reference level of 5 μg/dL, and the pre-2012 CDC “level of concern” of 10 μg/dL, were calculated for Flint, Detroit, and Michigan for the years 2011-19 using a dataset with 1,445,808 blood lead levels of all Michigan children tested, obtained from the Michigan Department of Health and Human Services (MDHHS) through a Data User Agreement (#202103-144) following IRB approval (IRB #202103-04-NR). Separately, de-duplicated data were also provided to us after MDHHS epidemiologists extracted the highest blood lead values per child per year using the following standard criteria (in order of preference):

- The highest venous blood lead test result available during the calendar year
- If there is no venous test result available, the highest capillary blood lead test result available during the calendar year
- If there is no test result with blood type available, the highest test result available during the calendar year

### Educational Outcomes

The data on all special education outcomes and general education 3rd grade reading proficiency for students enrolled in Flint Community Schools, Detroit Public Schools Community District, and all public schools in Michigan for the academic years 2011-12 until 2019-20 (or, latest available) were downloaded from the Michigan Department of Education’s website www.mischooldata.org. The special education enrollment data during 2006-07 to 2010-11 was obtained through Freedom of Information Act requests to the Michigan Department of Education.

### Poverty and Nutrition

The rates of poverty and households with children aged 0-18 years receiving food assistance (i.e., on Supplemental Nutrition Assistance Program) for Flint, Detroit and Michigan for 2011-19 were obtained from the US Census Bureau (US Census Bureau, 2022).

### Media Coverage and Social Media Interactions

A representative list of national and local media stories on lead exposure and educational outcomes of Flint children and Detroit children during October 2015-January 2021 (Table S1) was gathered using Google searches with keywords “lead”, “children”, “education”, “Flint” with and without the term “-Detroit” (i.e., removes all search results with “Detroit”), and “Detroit” with and without “-Flint”. The CrowdTangle extension v3.0.29 in Google’s Chrome Browser was utilized to gather total “interactions” (reactions, comments, and shares) of all Facebook users and total follower counts of public pages (e.g., celebrities, news organizations, and politicians) and public groups who shared the media stories on Facebook from publishing date until the time of conducting research (August 2020-September 2021). The representative negative expectations commentary of community leaders, teachers, parents and schoolchildren about lead exposure during the FWC period and educational difficulties for Flint and Detroit (Table S2) were gathered through manual screening of articles, posts and videos published during October 2015-January 2021, which were in turn obtained through open-ended Google searches using multiple keywords, including “Flint” (Flint only), “Detroit” (Detroit only), “Flint Water Crisis” (Flint only), “lead”, “poisoning”, “education”, and “children”. Separately, the total count and number of interactions data for all posts and web links shared on official Facebook pages of Michigan local media (Data S1) with the keywords “lead poisoned” during January 2016-November 2020 were downloaded from CrowdTangle (www.crowdtangle.org) and network maps were plotted in Gephi v0.9.2 (CrowdTangle, 2020).

### Statistical Analyses

All analyses were conducted in Excel® 2016 (Microsoft), SAS® 9.4 (SAS Institute, Cary NC), or GraphPad PRISM 8.4.3 (GraphPad Software). General linear mixed-effects modeling was used to model changes in binary effects over time, between Flint and Detroit (both nested within Michigan). Data are presented as mean with 95% confidence intervals. Pairwise planned comparisons sliced through each year were made and *p* < .05 was considered significant after false discovery rate adjustment within each outcome. All tests were two tailed. No further adjustment for multiple comparisons was performed. Ordinary least squares regression lines were fitted between log of percentage students enrolled in special education and log %EBL in the same years and the slopes compared within GraphPad PRISM.

## Results

This retrospective population-based cohort study utilized longitudinal datasets (de-identified aggregated yearly data) for Flint, the control city Detroit, and the entire State of Michigan to examine the hypothesized link between lead exposure and educational outcomes.

### Elevated Blood Lead

**Figure 1:**
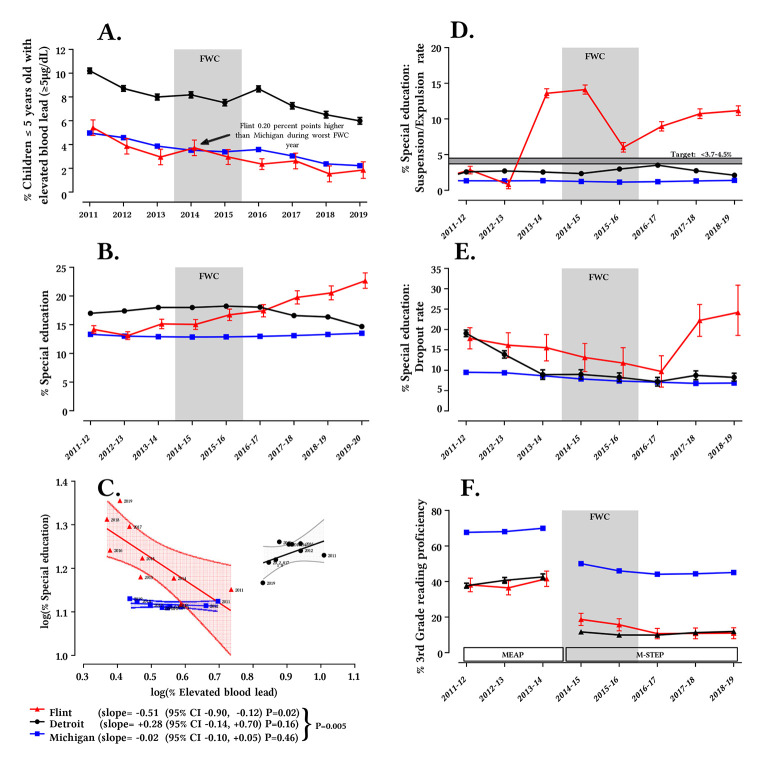
Childhood Blood Lead and Educational Outcomes *Note.* Trends in **(A)** percentage of children < 6 years of age with elevated blood lead ≥ 5 μg/dL (%EBL), **(B)** enrollment of public school students in special education programs, **(D)** special education suspension/expulsion rates, **(E)** special education dropout rates, and **(F)** general education 3rd grade reading proficiency*, for Flint, Detroit, and Michigan, 2011-19 (and corresponding school years of 2011-12 to 2019-20). Error bars are +/- 95% confidence intervals and maybe contained within symbols. **(C)** Scatter plot between %EBL vs. special education enrollment rate for Flint, Detroit, and Michigan by year. 95% confidence bands for the ordinary least squares fits are shown. *p* value shown is for comparison of slopes. *The State of Michigan followed the Michigan Educational Assessment Program (MEAP) testing standards until 2013-14 and then switched to Michigan Student Test of Educational Progress (M-STEP) starting 2014-15.

The proportion of children < 6 years with elevated blood lead (%EBL) at or above the 5 μg/dL CDC reference level decreased significantly from 2011 to 2019, *p*_(time)_ < .0001, in Flint, Detroit and Michigan overall. The %EBL in Flint steadily decreased by 65.8% between 2011-19 from 5.42% to 1.85%, Risk Ratio = 0.43, 95% CI [0.33, 0.56], *p* < .0001, notwithstanding the FWC increase that occurred in the months of June-August 2014 immediately following the water switch (1,17). The corresponding %EBL in Detroit and Michigan also saw large decrements of 41.3%, Risk Ratio = 0.69, 95% CI [0.65, 0.74], *p* < .0001) and 55.3%, Risk Ratio = 0.58, 95% CI [0.56, 0.61] from 2011-2019, respectively (Figure 1a).

There were also substantial differences in %EBL between Flint, Detroit and all of Michigan (Figure 1a) (*p*_(time*center)_ < .0001). Specifically, %EBL in Flint was 47-77% lower than for Detroit during 2011-19. Even in the worst FWC year of 2014, children in Detroit had more than double the %EBL of Flint. The %EBL in Flint (which comprised 2.2% to 2.4% of the State population) was also 13-35% lower than for the State of Michigan between 2012-19, with the exception of 2014 when Flint exceeded the %EBL in Michigan by 0.20 percentage points (i.e., 3.72% in Flint vs. 3.52% in Michigan). In other words, the net effect of the FWC, was to temporarily raise the blood lead of Flint children, up to the average for all data reported by the State of Michigan.

The relative trends between Flint, Detroit, and Michigan at the pre-2012 CDC 10 μg/dL “level of concern” blood lead threshold (%EBL10) were somewhat similar (Figure S1) to those seen at the 5 μg/dL level (%EBL). The %EBL10 for Flint was statistically indistinguishable from Michigan during 2011-19 even during the 2014 and 2015 FWC years. Finally, the %EBL10 for Flint was 65-77% lower than Detroit (*p* < .00001) for the entire 2011-19 time period.

**Figure 2:**
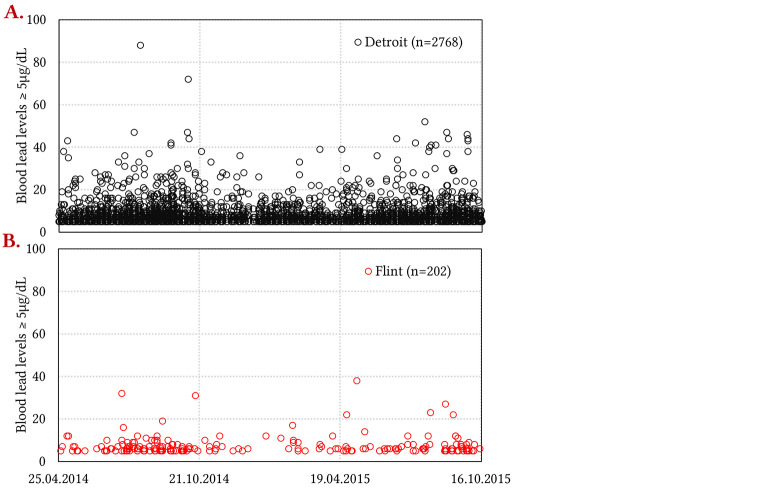
Individual Child-Level Blood Lead Measurements ≥ 5 µg/dL During the FWC Period (April 25 2014-October 16 2015) *Note.* **(A)** Detroit and **(B)** Flint. The data is de-duplicated; i.e., only highest blood lead value per child is shown.

**Figure 3:**
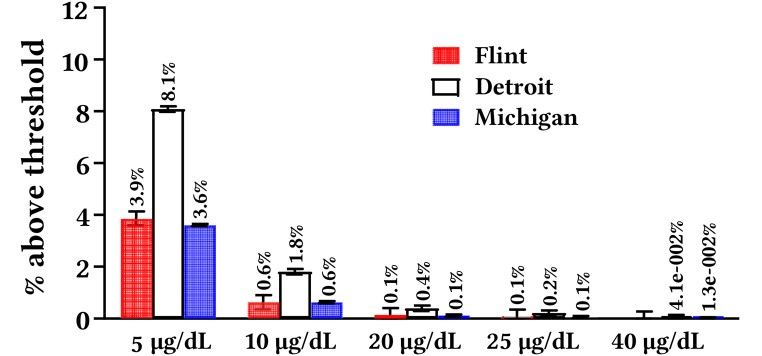
Percentage of Children < 6 Years of Age With Blood Lead ≥ 5 – 40 µg/dL in Flint, Detroit, and Michigan During April 25, 2014 – October 16, 2015 *Note.* The CDC threshold for elevated blood lead was 40 µg/dL between 1973-75, 25 µg/dL between 1985-90, 10 µg/dL until 2012 and 5 µg/dL until 2021.

Analysis at the individual child-level was conducted to consider isolated cases of anomalously high blood lead from acute exposure during the FWC that were possibly masked by yearly aggregated trends (i.e., Figure 1a). Plotting blood lead measurements of every tested child in Flint and Detroit with blood lead ≥ 5 µg/dL (Figure 2a-2b) and comparing the count and percentage of children in Flint, Detroit, and Michigan with blood lead ≥ 5, 10, 20, 25 and 40 µg/dL (Figure 3) during the FWC period of 539 days (April 25 2014-October 16 2015) revealed:

- The mean blood lead of all children with blood lead ≥ 5 µg/dL in Detroit was 12.4% higher than in Flint (unpaired two-tailed *t*-test; *p* < .05).
- The count of Detroit children was higher than Flint children in every blood lead category (≥ 5-40 µg/dL).
- Detroit children had statistically higher blood lead than Flint children at and above the 5 and 10 µg/dL blood lead thresholds.
- Data for Flint children were statistically indistinguishable from that reported for all State of Michigan children in every blood lead category (≥ 5-40 µg/dL).
- There were 28 children who tested at or above 40 µg/dL in Michigan during the FWC period, of which half (14) were in Detroit and none (0) in Flint.
- There were four Flint children with blood lead at or above 25 µg/dL, both during the FWC and in the same time duration pre-FWC (November 1 2012 – April 24 2014).

### Overall Educational Outcomes

Of 23 special education outcomes monitored each academic year, nine worsened, nine improved and five did not change (≤±1% change) in Flint after the crisis vis-à-vis before the water crisis (see Text S2, Supplementary Materials). In a simple comparison relative to Detroit, only three outcomes worsened and another three improved in Flint.

Despite these overall neutral trends, four worsening outcomes in Flint were nonetheless emphasized and attributed to lead exposure from the FWC by the national media and experts (ACLU, 2016; Alfonsi, 2020; Green, 2019; Redlener, 2018; Riley, 2018; Strauss, 2019) including: a) special education enrollment; b) suspension or expulsion for children in special education; c) dropout for children in special education, and d) worsening reading proficiency of 3^rd^ grade students in general education.

**Figure 4:**
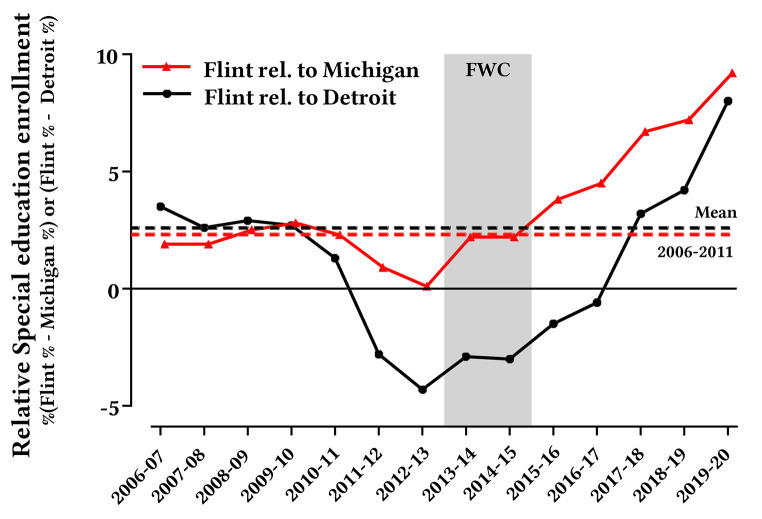
Special Education Enrollment in Flint Relative to Detroit and Michigan, 2006-20

Each of these attributions is examined in greater detail for Flint Community Schools, Detroit Public Schools Community District, and all public schools in Michigan using data for the academic years 2011-12 until 2019-20. Special education enrollment was also examined from 2006-07 onwards to identify a baseline (Figure 4), and 3^rd^ grade reading proficiency was not analyzed in 2019-2020 since tests were cancelled due to the COVID-19 pandemic.

#### Special Education Enrollment

Special education enrollment trends are routinely gathered under federal and state laws (Individuals with Disabilities Act, 2004; Michigan Department of Education, 2020; Weiss & Mettrick, 2010), and the data are significantly different for Michigan, Detroit and Flint (*p* < .0001, Figure 1b). Overall, in the State of Michigan, the proportion of children in special education remained stable between 2011-12 and 2019-20 at 12.9-13.5%. The special education enrollment rate in Detroit slightly increased from 2011-12 to 2016-17 followed by a downtrend during 2017-18 to 2019-20 (Figure 1b). The special education enrollment rate in Flint started lower than Detroit (*p* < .0001) in 2011-12 as would be expected due to lower blood lead levels alone, but began rising in 2015-16 (relative to the previous three academic years) after the federal emergency and aggressive national and international reporting on the FWC (Green, 2019; Jackson, 2017; Pew Research Center, 2017). Flint special education enrollment even surpassed that in Detroit in 2017-18 (*p* < .0001). Notably, the spike in Flint special education enrollment rate only occurred in the 6-21 year age group, whereas the age group that would be considered most vulnerable to water lead exposure (i.e. those in the womb or up to age 1 during the FWC) saw no significant increase (Figure S2).

The special education enrollment rate in Flint relative to Michigan during 2013-15, including the first FWC year, was comparable to the 2006-11 baseline, but began to spike in the second FWC year (2015-16), when media coverage on the crisis increased markedly (see Figure 4, “Flint relative to Michigan”). This was associated with a strong diverging trend between the special education rates for Flint and Detroit starting in 2016-17 (Figure 1b). Similarly, Flint special education enrollment was much lower relative to Detroit between 2011-16 (see Figure 4, “Flint relative to Detroit”), became comparable in 2016-17, and increased dramatically from 2017-20. There is actually a strong inverse relationship (Figure 1c) between %EBL and special education enrollment rate in Flint, *r* = -0.79, 95% CI [-.96, -0.18], *p* = .021, but there is no such relationship for the same time period in Detroit, *r* = 0.20, 95% CI [-0.59, 0.79], *p* = 0.63, or Michigan, *r* = 0.09, 95% CI [0.66, 0.75], *p* = 0.83).

#### Special Education Suspension/Expulsion Rates

The special education suspension/expulsion rates in Flint increased 7.4 times in 2013-14 (13.6%) before the FWC began (Figure 1d) compared to the previous two school years, and peaked in the first FWC year 2014-15 (14.1%), before dropping more than half in the second FWC year 2015-16 (6%). Rates progressively rose during 2016-19 (9% to 11.2%) after the FWC came to light.

#### Special Education Dropout Rates

The special education dropout rates in Flint roughly doubled in the 2017-20 school years (22.1%) versus 2014-17 (11.5%), after a steady decline during 2011-17 analogous to that occurring in Detroit and Michigan (Figure 1e).

#### General Education Reading Proficiency

After the State of Michigan adopted the stricter Michigan Student Test of Educational Progress (M-STEP) standard in the 2014-15 school year, both Flint (22.3 percentage points) and Michigan (19.9 percentage points) witnessed identical drops (around 20 percentage points) in 3rd grade reading proficiency between 2013-14 and 2014-15, but Detroit fell even more precipitously (31 percentage points) (Figure 1f). During the FWC (2014-16 school year) and until the 2018-2019 school year, 3rd grade reading proficiency stayed roughly the same in Detroit, however Flint continued to decrease until reaching the same level as Detroit.

### Potential FWC Covariates That Could Explain Rising Special Education Enrollment

Analyses of trends in covariates including waterborne contaminants besides lead, poverty, poor nutrition, City of Flint’s administration and emergency management decisions, and Flint Community Schools’ policies and funding do not appear to be primarily associated with the post-FWC rise in special education enrollment in Flint (see Text S1, Supplementary Materials).

#### Negative Community Expectations and Media Coverage

The aggregated data from Facebook, the dominant social media platform in the United States (Perrin & Anderson, 2019), obtained using the CrowdTangle (www.crowdtangle.com) public insights tool owned and operated by Facebook, indicate news around lead “poisoning” of Flint children and worsening educational outcomes was interacted with hundreds of thousands of times and potentially reached tens of millions of users on Facebook alone between 2015-21 (Table S1). In contrast, there were just two articles about Detroit children that saw just over 22,000 interactions. On average, 12.2% web traffic to news websites originate from social media, and, therefore, these reported values are gross underestimates of the total “reach” of news, which would include all newspaper and magazine hard copies read, news channel broadcasts watched, and radio programs and podcasts heard (Alexa, 2020). To illustrate, the *60 Minutes* Flint special education episode (Alfonsi, 2020) alone was interacted with over 27,000 times and potentially reached ~10 million users on Facebook, and its television broadcast was also watched by over 10 million viewers on the *CBS* channel (Table S1). Negative pronouncements about lead exposure during the FWC period and educational difficulties in the media disproportionately originated from Flint community leaders describing Flint children, but similar claims were not made publicly by Detroit community leaders (Table S2) despite the much higher blood lead for Detroit children (Figure 1a, Figure 2a, 2b, Figure 3 and Figure S1).

**Figure 5:**
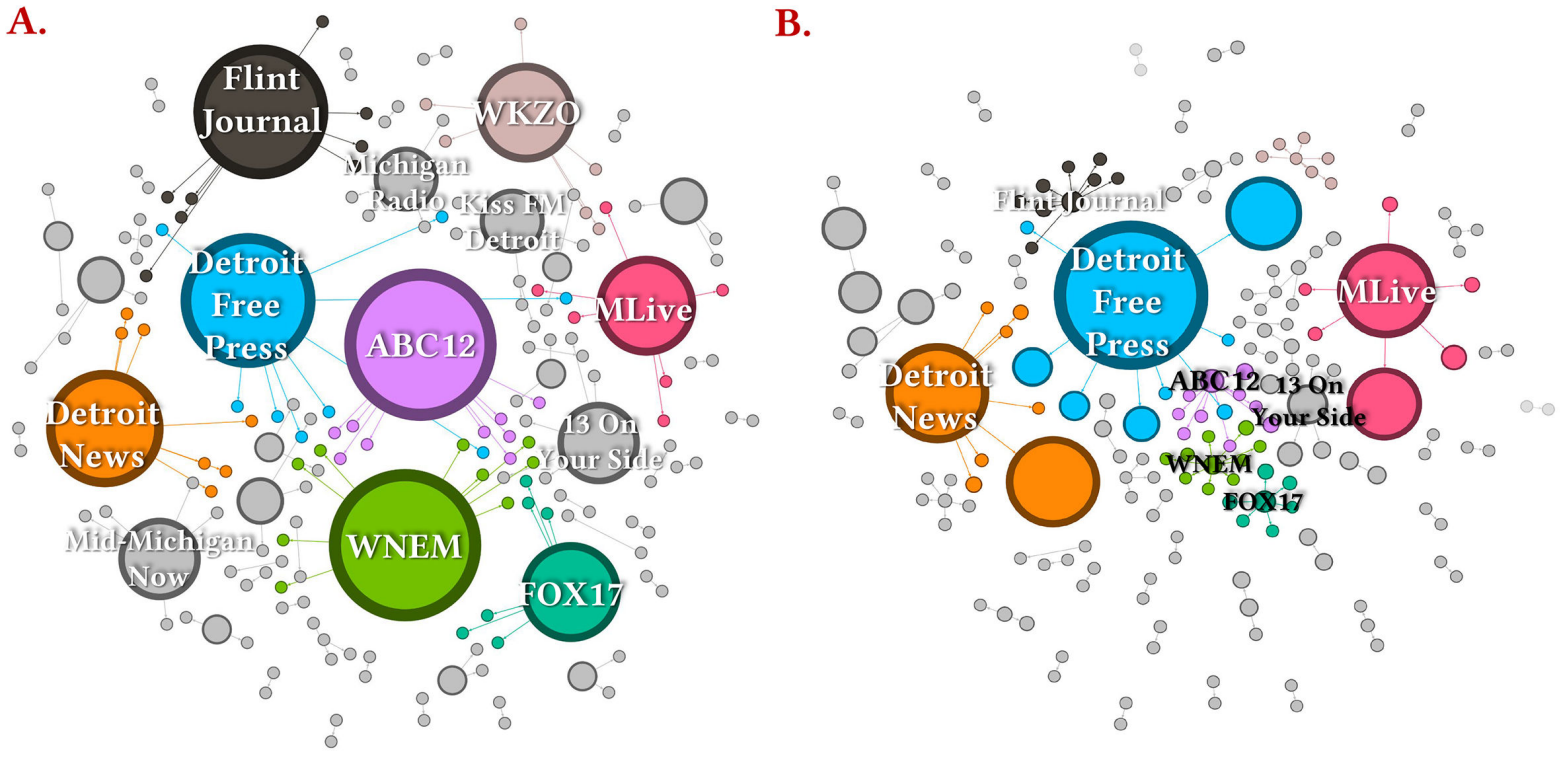
Network Mapping of All Posts and Weblinks Shared on Official Facebook Pages of Michigan Local Media With the Keywords “Lead Poisoned,” Jan 2016-Nov 2020 *Note.* **(A)** Media outlets arranged by total number of posts/links shared. **(B)** Media outlets arranged by total interactions (reactions, shares, and comments) on posts/links shared. Raw values for all media bubbles in the maps are provided in SI (Table S4). **(i)** The size of the media bubbles is relative; i.e., higher the metric of interest, larger the bubble. The numerous links emerging from each bubble indicate resharing of the posts/links to other Facebook pages and the representative bubbles are also relatively sized according to the metric of interest. **(ii)** The Flint Journal belongs to the parent media company MLive and both have separate Facebook pages. Therefore, while the pages appear separately in the left network map as they do in Facebook, their interaction metrics are aggregated in text.

A search for all posts and weblinks shared on official Facebook pages of Michigan local media (Data S1) with the keywords “lead poisoned” and no mention of “Flint” or “Detroit” during January 2016-November 2020 time period using CrowdTangle revealed over 80% articles (Data S2) discussed the FWC. Network mapping of these posts (Figure 5) revealed the media outlets who shared the most articles and links to be from Flint (*WNEM TV5*, *ABC12*, and *The Flint Journal/MLive*; collective *n* = 32) followed by prominent state-level newspapers *Detroit Free Press* and *The Detroit News*. Moreover, the postings from Michigan’s Top three dailies by circulation (i.e., *Detroit Free Press*, *The Flint Journal/MLive*, and *Detroit News*) (Agility PR, 2020) saw the most interactions (~35,000) in the form of reactions, shares, and comments.

## Discussion

This investigation confirms that the proportion of young children with elevated blood lead in Flint, Detroit and Michigan as a whole has been declining over the past 10 years. The %EBL trend in Flint is very similar to that observed across Michigan and has always been much lower than Detroit. In fact, the %EBL in Flint has now dropped below data for the United States (US Centers for Disease Control and Prevention, 2020).

For additional perspective, the geometric mean blood lead even during the worst FWC year as reported in previous research (Gómez et al., 2018), was lower than that reported in the European nations of France and Poland (Table S3). Paradoxically, since the FWC was revealed in 2015 and residents were further protected from exposure to waterborne lead, Flint saw a dramatic spike in special education enrollment, while such enrollment remained steady across Michigan and even declined after 2015-16 in the control city of Detroit.

While lead is a neurotoxin with known potential for worsening educational outcomes (Jusko et al., 2008; Mendelsohn et al., 1998; Surkan et al., 2007; Watt et al., 1996), analysis of the data in Flint relative to Detroit is inconsistent with the attribution of rising special education enrollment in Flint to lead exposure. The worst lead exposure from the FWC was of relatively short duration (about one-sixth of the entire time on Flint River water; Roy et al., 2019), and is set against a historic decline in blood lead in Flint as well as Detroit, Michigan and nationally (Dignam et al., 2019; Gómez et al., 2018). The elevation in Flint childhood blood lead was above the relatively new 5 μg/dL CDC reference threshold but not the 10 μg/dL threshold “level of concern” exceeded in Washington DC children during its 2001-04 lead in drinking water crisis (Roy et al., 2019). The number of individual children testing ≥ 25 μg/dL, a threshold above which it is reported that 20% of children require an average of nine years of special education (Swinburn, 2016), was 18 times higher in Detroit (0.21% of all children tested; *n* = 73) than in Flint (0.08% of all children tested; *n* = 4) during the FWC.

As early as January 2016, it was acknowledged that the worst-case incidence of elevated blood lead during the FWC was always less than half of the incidence in other Michigan cities of Detroit, Grand Rapids, and Muskegon, and 3,800 other communities across the United States (Frazier, 2018; Lanphear, 2017a; Mack, 2016; Pell & Schneyer, 2016; Wilkinson, 2016). Moreover, since %EBL in Detroit was always at least twice that in Flint before, during and after the FWC, worse outcomes, whether concurrent or lagged, would always be expected for Detroit children, but such an impact is not observed in the time period of interest. Instead, there is an incongruous inverse relationship between childhood blood lead and special education enrollment in Flint, while no such relationship exists for Detroit and Michigan.

Despite an equal number of overall special education outcomes worsening and improving (see Text S1, Supplementary Materials), only those that superficially appeared to be worsening were publicized in the media. Our detailed analysis shows these outcomes are insignificant or inconsistent with the actual lead exposure that occurred. Specifically, the seven-fold jump in suspension/expulsion rates of special education students had occurred in 2013-14 before the onset of the crisis, and the comparison with Detroit further discounts an association with lead exposure. Indiscriminate enforcement of suspension/expulsion policies before the FWC (D.R. v. Michigan Department of Education, 2016) may have contributed to this spike. The special education dropout rates in Flint only started to rise in 2017-18 post-FWC after expectations of such an outcome was widely publicized in the media starting late 2016. Finally, the reduction in Flint general education 3rd grade reading proficiency after adoption of a new academic standard in 2014-15 was also observed in the Detroit control group, and could be attributed to the changed tests.

The rise in special education enrollment in Flint following the FWC was not associated with confounders of waterborne contaminants besides lead during the FWC, poverty, poor nutrition, and emergency management. The Flint schools’ failure to properly enforce special education policies and a severe budget deficit since the early 2010s may have contributed to less Flint students being enrolled in special education programs pre-FWC, but the enrollment rate had returned to historical norms during the FWC.

A nocebo effect is consistent with the trend of rising special education enrollment after the FWC was exposed (Colloca & Barsky, 2020; Petrie & Rief, 2019). As a top news story of 2016, the crisis engendered negative psychological effects described by residents as “Flint fatigue,” and the surrounding international media coverage has continued for over five years with negative headlines (Adams, 2016; Associated Press, 2020; Cuthbertson et al., 2016; Goodnough & Atkinson, 2016; Heard-Garris et al., 2017; May, 2016). The news reports and their popularity on social media (Table S1, Figure 5) and negative perceptions of Flint community leaders and parents (Table S2) could have heightened negative expectations about the effects on children, who readily accept and act on information from those they trust (Harris & Corriveau, 2011; Jaswal et al., 2010; Landrum et al., 2013). Contaminated water creates high public anxiety compared to other environmental concerns (Petrie et al., 2001). For example, the psychological impact of the FWC caused increased tap water avoidance amongst US children nationwide after the FWC came to light (Rosinger & Young, 2020). The early speculation and worst case predictions of impacts on Flint children were also made in a vacuum of trust, uncertainties in the timing and magnitude of the water lead exposure due to manipulation of official test results, and an acknowledged “failure of government at all levels” that caused the FWC (Roy & Edwards, 2019a).

From 2016-18 arguments over the possible negative consequences of labeling Flint children “poisoned” versus “exposed” played out in the media (Clark & Filardo, 2018; Drum, 2017; Gómez & Dietrich, 2018; Mays, 2018; Schneider et al., 2016; Shell, 2016). The worst negative expectations for special education enrollments in years following the FWC appear to have been realized, even though comprehensive blood and water lead analyses eventually published in 2018-20 (Gómez et al., 2018; Gómez et al., 2019; Roy et al., 2019; Roy & Edwards, 2020) contradict the popular belief that Flint children experienced an unprecedented environmental lead exposure (Figure 1a). Moreover, in many cases, the national media – e.g., *The New York Times* (Green, 2019) and *CBS 60 Minutes* (Alfonsi, 2020) – have provided even worse prognoses, labeling Flint children as brain damaged or lead poisoned (Table S1). No comparable media labeling was applied to children in Detroit (Table S1, Figure 5) or the other Michigan cities with much higher %EBL incidence. A significant percentage of Flint households experience water crisis-related stress and other negative psychological effects, are meeting criteria for psychological trauma, report behavioral problems in their children, and believe that “the crisis would never be fixed” (Bosman & Greeson, 2020; Brooks & Patel, 2022; Ezell & Chase, 2021; Jones et al., 2022; Reuben et al., 2022; Sneed et al., 2020; Trejo et al., 2022).

A perception that Flint’s water is still unsafe and a source of ongoing community concern is supported by continued high rates of bottled water use five years after the switchback to Detroit water. Bottled water use has persisted despite distribution of free lead filters, replacement of over 90% of lead pipes, and independent tests showing current Flint water lead levels to be lower than observed in other Michigan cities with old pipes (Alfonsi, 2020; City of Flint, 2022; Flint Cares, 2018; Reuben et al., 2022; Roy & Edwards, 2019a, 2020). In fact, it is reported that some of Flint’s youngest children have only bathed in and consumed bottled water their entire lives (Alfonsi, 2020; Fonger et al., 2019; Herndon, 2018).

Exposure to feared contaminants such as lead is known to create nocebo responses (Blettner et al., 2009; Crichton et al., 2014; Gruber et al., 2018; Petrie et al., 2005; Small & Borus, 1987; Witthöft & Rubin, 2013). Other suspected water contamination incidents have caused health complaints that were difficult to explain by the level of toxicological exposure (David & Wessely, 1995; Page et al., 2006). In the Camelford water contamination incident in Cornwall, England, health complaints were intensified by media interest, concerns about a conspiracy and litigation (David & Wessely, 1995). However, in contrast to the FWC, those studies did not have direct data from continuous monitoring of the contaminant of concern in the blood of the affected population, or suitable control groups for comparison as in the research results presented herein.

It has also been argued that the actual (and small) magnitude of elevation in children’s blood lead from the FWC does not matter in terms of the resulting health harm (e.g., Hanna-Attisha et al., 2018; Kuehn, 2016; Oleske et al., 2016; Schmidt, 2018; Schneider et al., 2016; Stateside Staff, 2018). However, the epidemiological study by Lanphear and colleagues noted an inverse, supralinear dose-response relationship: a net decrease of 6.9 IQ points, 95% CI [4.2, 9.4] for blood lead increment of 2.4 to 30 µg/dL, with the steepest drop of 3.9 IQ points, 95% CI [2.4, 5.3] occurring for the lowest blood lead range of 2.4 to 10 µg/dL (Lanphear et al., 2005). While the underlying (biological) mechanism has not been elucidated (Lanphear, 2017b), the supralinear curve confirms the scientific principle that “the dose makes the poison” for lead.

These data suggest that the rising enrollment in special education attributed to the FWC, may be associated with widespread negative expectations and not an elevation in blood lead. This possible nocebo effect in Flint represents an unfortunate natural large-scale experiment, in which a population has been repeatedly informed by trusted national and international media sources that an unprecedented lead exposure event had occurred with severe long-term adverse repercussions to children, even when the data indicate that the actual lead exposure was normal for the State and less than nearby communities.

Two of this paper’s authors (MAE/SR) personally witnessed such expectations during a science outreach program for over 1,000 K-12 Flint students in March 2017 (Edwards, 2017; Jacques, 2018), where several teachers openly expressed their belief that Flint children had been brain damaged, were incapable of learning, and that there was little point in trying to teach them (Bouffard, 2018; Edwards, 2017; Jacques, 2018; Roy & Edwards, 2019c). Trust of teachers in students and parents is a significant predictor of student achievement (Goddard et al., 2001). These and similar expectations have been broadcast in the media for over five years (e.g., Tables S1 and S2, Figure 5) and can strongly influence children’s school performance and behavior, such as those previously documented in younger and stigmatized children from African-American and lower socioeconomic backgrounds (Jussim et al., 1996).

Students who require and receive special education services do benefit from them (Ballis & Heath, 2021) and higher special education enrollments are not necessarily indicative of permanent brain damage or health harm from the water lead exposure. In fact, part of the rise might be viewed as part of a proactive effort to compensate for the failures of government at all levels that caused the FWC (Wagner & Kennedy, 2016). In any event, the media have never publicized this possible positive interpretation.

Importantly, the media messaging has not changed, in spite of ample evidence that the actual lead exposures in Flint were not abnormally high relative to all of Michigan and were much lower than neighboring Detroit. It is possible that the harm from such messaging is continuing. For instance, the special education enrollment rate in Flint for 2019-20 (22.7%) is now over 1.5-1.7 times the rates in Detroit (14.7%), and is higher than Michigan (13.5%) and the United States overall (14.1%) (US Department of Education, 2020), despite the fact that Detroit children have always had more than double the incidence of %EBL than Flint (Figure 1a). This trend may even be accelerating due to universal special neuropsychological screening now being conducted for Flint children, which has recently indicated an 80% diagnosis rate for “language, learning or intellectual disorders'' that are attributed to lead exposure from the FWC (Alfonsi, 2020; Chambers, 2019).

Our study has limitations. This study is limited by reliance on existing blood lead datasets collected under standard screening practices, covering about 23% of young children in Michigan, 40% in Detroit and 39% of Genesee Co. in 2016 (Michigan Department of Health and Human Services, 2018). Since this is a population-based study, we did not have educational outcomes data at the individual level to adjust for potential confounders or to identify if multiple adverse outcomes were occurring for the same children. Our study is also limited by the lack of charter school data. And finally, our central analyses were correlational, and should be interpreted with caution. In contrast, the strength of this population-based study is the utilization of over 1.44 million individual childhood blood lead measurements and annual monitoring of outcomes in general and special education occurring under uniform Michigan educational policies in two cities with comparable demographics, using the same source of treated drinking water from Lake Huron except for the 18 months Flint was served by the Flint River and suffered the manmade public health crisis. The educational outcomes data are representative as they are weighted by city-level population instead of individual schools. The novel contribution of this study is uncovering of possible nocebo effect in the aftermath of a public health emergency involving a known neurotoxin, via an unfortunate natural experiment that could never have been studied intentionally.

## Supplementary Materials

The Supplementary Materials contain the following items (for access see Index of Supplementary Materials below):

- Figures S1 to S2
- Tables S1 to S4
- Texts S1 (including Figure S3) to S2 (including Figures S4 to S22 and Table S5)
- References
- Data S1 to S2

RoyS.
PetrieK. J.
GambleG.
EdwardsM. A.
 (2023). Supplementary materials to "Did a nocebo effect contribute to the rise in special education enrollment following the Flint, Michigan Water Crisis?"
[Additional information]. PsychOpen. 10.23668/psycharchives.12578
PMC1010315837065004

## Data Availability

All education data are publicly available on Michigan Department of Education’s website www.mischooldata.org. Blood lead data were obtained from Michigan Department of Health and Human Services under a Data User Agreement (DUA #202103-144) following IRB approval (IRB #202103-04-NR). The data can be made available from MDHHS upon completion of a data use agreement with the agency. The authors assume full responsibility for the analysis and interpretation of the data. All poverty and food assistance data are publicly available from the US Census. All media coverage and Facebook interactions data downloaded from CrowdTangle are available in the Supplementary Materials.
